# Unveiling Distribution Patterns of Freshwater Phytoplankton by a Next Generation Sequencing Based Approach

**DOI:** 10.1371/journal.pone.0053516

**Published:** 2013-01-22

**Authors:** Alexander Eiler, Stina Drakare, Stefan Bertilsson, Jakob Pernthaler, Sari Peura, Carina Rofner, Karel Simek, Yang Yang, Petr Znachor, Eva S. Lindström

**Affiliations:** 1 Uppsala University, Department of Ecology and Genetics, Limnology, Uppsala, Sweden; 2 Swedish University of Agricultural Sciences, Aquatic Sciences and Assessment, Uppsala, Sweden; 3 Limnological Station, Institute of Plant Biology, Kilchberg, Switzerland; 4 University of Jyväskylä, Department of Biological and Environmental Science, Jyväskylä, Finland; 5 Biology Centre of the Academy of Sciences of the Czech Republic, v.v.i., Institute of Hydrobiology, České Budějovice, Czech Republic; 6 Uppsala University, Erken Laboratory, Norrtälje, Sweden; University of Nottingham, United Kingdom

## Abstract

The recognition and discrimination of phytoplankton species is one of the foundations of freshwater biodiversity research and environmental monitoring. This step is frequently a bottleneck in the analytical chain from sampling to data analysis and subsequent environmental status evaluation. Here we present phytoplankton diversity data from 49 lakes including three seasonal surveys assessed by next generation sequencing (NGS) of 16S ribosomal RNA chloroplast and cyanobacterial gene amplicons and also compare part of these datasets with identification based on morphology. Direct comparison of NGS to microscopic data from three time-series showed that NGS was able to capture the seasonality in phytoplankton succession as observed by microscopy. Still, the PCR-based approach was only semi-quantitative, and detailed NGS and microscopy taxa lists had only low taxonomic correspondence. This is probably due to, both, methodological constraints and current discrepancies in taxonomic frameworks. Discrepancies included Euglenophyta and Heterokonta that were scarce in the NGS but frequently detected by microscopy and Cyanobacteria that were in general more abundant and classified with high resolution by NGS. A deep-branching taxonomically unclassified cluster was frequently detected by NGS but could not be linked to any group identified by microscopy. NGS derived phytoplankton composition differed significantly among lakes with different trophic status, showing that our approach can resolve phytoplankton communities at a level relevant for ecosystem management. The high reproducibility and potential for standardization and parallelization makes our NGS approach an excellent candidate for simultaneous monitoring of prokaryotic and eukaryotic phytoplankton in inland waters.

## Introduction

Phytoplankton are essential for biogeochemical cycles [1] and form the base of aquatic food webs [2], [3]. Their excessive growth can also cause significant threats to local biodiversity and ecosystem functioning, as in the case of toxic algal blooms [4]. Consequently, phytoplankton are used to monitor the status of aquatic ecosystems and there is a need to understand and predict the responses of these communities to shifting environmental conditions, such as climate change, increasing nutrient inputs, and modifications in flow regimes and land use due to an increasing anthropogenic pressure [4], [5]. Considering that phytoplankton species differ widely in nutrient requirements, susceptibility to predation and toxicity, it is important to understand not only the drivers of total phytoplankton biomass but also of their community composition.

So far, most studies on the diversity, distribution, and abundance of phytoplankton taxa have been based on morphological characteristics using different microscopic techniques. There are so far no studies on monitoring of combined phytoplankton communities (i.e. both cyanobacteria and eukaryotic algae) with molecular methods, but separate monitoring of eukaryotic phytoplankton communities have been attempted using single-strand conformation polymorphism and microarrays [6], real-time PCR (targeting toxic *Alexandrium* sp.) [7] and terminal restriction fragment length polymorphism [8], [9]. Recently, the development and throughput of DNA sequencing technology in the form of next generation sequencing (NGS) has taken giant leaps forward [10], [11]. These developments have facilitated extensive sequence-based characterization of diverse natural microbial communities. Compared to microscopy, there are multiple advantages of using DNA-sequencing for analysis of phytoplankton communities. For instance, sample handling and preparation can be automated and thereby lower analytical costs as well as increase speed of analyses. This makes it possible to increase sampling frequency across both time and space and facilitate large scale comparisons of results from very different aquatic systems. By using the same protocol, it is also possible to standardize the analyses in different laboratories around the globe. Since this sequence-based taxonomic identification can be done in an identical way regardless of operator and laboratory, this significantly improves the potential for cross-system comparisons. Microscopic identification on the other hand, relies heavily on the skills and experience of each taxonomist. This may lead to consistency problems when more than one operator carries out the analyses, for instance in long term water monitoring projects or global comparative studies, as taxonomic resolution is quite likely to vary. Another advantage of molecular approaches is that it becomes possible to recognize and identify nano- and picophytoplankton that cannot be discriminated based on morphological features, such as unicellular cyanobacteria and small flagellates [12]. Furthermore, NGS based approaches allow the accurate identification of rare and fragile phytoplankton taxa, allow unmasking of look-a-likes and do not discriminate between life stages. A final advantage is the fact that evolving sequence-based phytoplankton monitoring datasets can be re-analyzed at a later time, using more refined taxonomic reference databases and other new information.

In the aquatic environment, these new sequencing technologies have already been introduced in studies on the diversity of other organisms lacking morphological detail for identification e.g. bacteria [13]–[17], archaea [18], [19] and microeukaryotes [20]. NGS is now allowing us to study patterns of microbial diversity in much greater detail than with microscopy or previously used molecular techniques [10], and should be equally useful for phytoplankton communities. However, the choice of the most informative taxonomic marker gene is still highly debated for phytoplankton and has so far hindered the large scale application of NGS facilitated approaches for phytoplankton monitoring. Still, the NGS method itself is global as it can be applied to every taxonomic marker with appropriate PCR primer sites and hence its development is independent from the marker of choice.

Here, we use the 16S rRNA gene as a marker as it is universal in prokaryotes including cyanobacteria and also universally present in the chloroplasts of eukaryotes. This enables simultaneous detection of prokaryotic and eukaryotic phytoplankton taxa. Using datasets based on 16S rRNA gene amplicons that have been sequenced by 454 pyrosequencing, we describe temporal patterns in three lakes and compare phytoplankton communities among an additional 46 lakes from temperate, boreal and polar regions. Our sequence-based data reveals that phytoplankton composition differ significantly among lakes with different trophic status showing that our approach can resolve phytoplankton communities and act as a tool for monitoring trophic status of aquatic systems. Our study illustrates the potential of DNA sequencing-based analyses as powerful tools in environmental monitoring by offering accurate, reliable and rapid identification of phytoplankton taxa from complex environmental samples.

## Methods

### Sampling

Water samples were taken from a range of lakes of different nutrient content (including also some saline Antarctic lakes) as described previously for Erken (ER [14]); Alinen Mustajärvi (AM), Mekkojarvi (MJ), Nimetön (N), Valkea Kotinen (VK) and Valkea Mustajarvi (VM) [17]; Åtvändtjärnen (AT), Bodsjön (Bod), Bustadtjärnen (Bus), Digernästjärnen (DT), Gravatjärnen (GT), Häggsjön (Hag), Hallåstjärnen (Hat), Hensjön (Hes), Holmtjärnen (Holm), Lång-Björsjön (LBS), Medstugusjön (MS), Öster-Noren (ON), Skalsvattnet (SV), Tännsjön (TS), Väster-Noren (VN) [16]; Alstasjön (AS), Åresjön (AS), Bredsjön (Bre), Fibysjön (Fib), Funbosjön (Fun), Hasselasjön (Has), Långsjön (LAS), Lille Jonsvatn (LJ), Lötsjön (LOS), Lumpen (LUM), Övre Långsjön (OLS), man-made Římov Reservoir (RI), Norrsjön (NS), Ramsen (RA), Ramsjön (RS), Ryssjön (Rys), Siggeforasjön (Sig), Strandsjön (Str), Valloxen (VA) and Zurich (ZU; this study); and Antarctic systems [14]. Metadata including physiochemical parameters were determined as described previously [14]–[17] and are summarized in Table S1. Time-series data were obtained from four systems; AM, ER, RI and ZU were represented by 44, 71, 48 and 33 samples, respectively. Most other systems (N = 41) were only sampled once, and 11 systems were sampled twice, bringing the total number of samples to 259 samples with 56 lakes represented.

### Microscopy analysis of phytoplankton community composition

Samples for assessment of phytoplankton abundance and biomass were preserved with Lugol's solution. This was done for time series data from AM, ER and RI. Phytoplankton were enumerated using inverted microscopes at 100–1000×magnification, after sedimentation of a known volume of sample in a counting chamber [21]. The mean algal cell dimensions were obtained for biovolume calculation using the approximation of cell morphology to regular geometric shapes [22]. Species composition was determined to the finest level possible (usually species). Some taxa were grouped into non-taxonomical groups due to few morphological characteristics visible with the chosen analysis method. Each time-series was analyzed by different taxonomists using national taxonomic monographs.

### DNA extraction, PCR amplification and sequencing

Genomic DNA extraction from filters (0.2 µm) was performed using the Ultra clean Soil DNA extraction kit as recommended by the manufacturer (MoBio, Laboratories, Solana Beach, CA, USA). Except for lakes AM, MJ, N, VK and VM a modified protocol originally described by Griffiths et al. was used [17], [23]. DNA from the Antarctic lakes was extracted using the Power soil kit (MoBio) and for lakes AS, AT, Bod, Bre, Bus, DT, Fib, Fun, GT, Hag, Has, Hat, Hes, Holm, LBS, LAS, LJ, LOS, LUM, MS, OLS, ON, NS, RA, RS, Rys, Sig, SV, Str TS, VA and VN the Easy DNA extraction kit (Invitrogen, Carlsbad, CA, USA) was used. PCR amplification was performed using general bacterial primers 341F (CCTACGGGNGGCWGCAG) and reverse primers 805R (GACTACHVGGGTATCTAATCC) with 454 adaptors and a sample-specific barcode on the reverse primer [13] under conditions described previously [14]–[16]. The amplicons were pyrosequenced with the 454 GS FLX system (454 Life Sciences, Branford, CT, USA) by different laboratories using both FLX and Titanium chemistry following procedures as described in detail previously [13]–[17].

### Sequence processing

Output from the sequencer in the form of SFF files together with a list of samples including their corresponding barcodes were used for the analyses. First, ambiguous sequences were removed from the data set including reads with low quality as inferred from their flowcharts and those that did not carry the exact primer sequence (reverse primer 805R) [13]. After reads had been sorted into samples based on the barcodes, they were denoised using AmpliconNoise Version 1.24 [24]. AmpliconNoise implements algorithms that remove PCR and 454 pyrosequencing noise as well as the chimera removal tool Perseus. This procedure resulted in almost 1.2 Million high quality reads of which almost 90,000 were annotated as cyanobacteria or chloroplasts using a naïve Bayesian classifier [25] and the taxonomy after Hugenholtz [26].

To obtain a higher taxonomic resolution than provided by the classifier, a representative sequence from each OTU was aligned in MOTHUR [27] using kmer for finding the template sequence and Needleman for aligning sequences against the SILVA106 small subunit rRNA gene database [28]. Aligned sequences were imported into ARB [29] and the quick parsimony option was used to add the aligned sequences to the small subunit reference tree included in SILVA106 database. In addition, a refined classification was performed using an in-house cyanobacterial/chloroplast database using the naïve Bayesian classifier [25]. This database is based on the 16S rRNA gene sequences of cyanobacteria and eukaryotic chloroplasts from well-characterized phytoplankton entries of the SILVA106 database.

### Statistical analyses

To assign phytoplankton reads into operational taxonomic units (OTUs) prior to ordination procedures, sequences were clustered based on 97% sequence similarity using UCLUST [30]. The perl script daisychopper.pl (available at http://www.genomics.ceh.ac.uk/GeneSwytch/Tools. html) [31] was used to resample a selection of 139 samples (including only chloroplast and cyanobacteria related OTUs) to 100 reads prior to statistical analyses. Samples with less than 100 chloroplast and cyanobacteria reads were excluded from further analyses.

All statistical analyses were conducted using R (http://www.R-project.org/) [32] and the vegan package [33]. Non-metric multidimensional scaling of a Morisita-Horn distance matrix (function metaMDS) was used to visualize dynamics in phytoplankton community structure (beta diversity) using an OTU abundance matrix based on all OTUs represented by at least 3 reads in the non-resampled data matrix (194 OTUs). Permutational MANOVA was used to determine significant differences among lakes of different trophic status. Oligotrophic, mesotrophic, eutrophic and dystrophic as well as Antarctic samples were placed into their respective categories based on previous ecosystem characterization in the literature [14]–[17]. The direct comparison of the NGS data with microscopic data (cell abundances and biovolumes) were done from three lakes by both Procrustes superimposition and Mantel's test [33]. The three systems were analyzed individually as microscopy was performed each by a different taxonomist.

## Results

### Taxonomic composition

**Figure 1:**
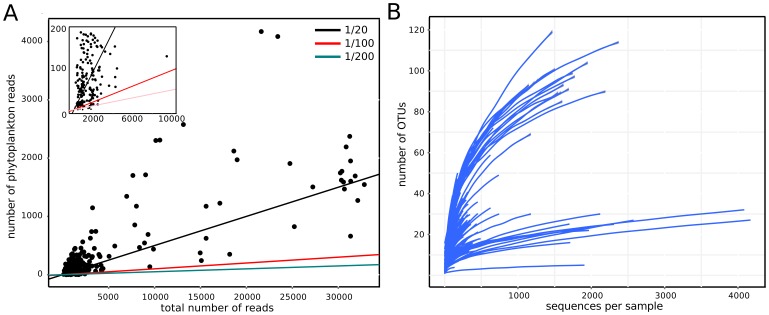
The ratio between the total number of reads and the number of phytoplankton reads (A) and rarefaction curves of the next-generation sequencing data (from all 259 samples) (B). The lines depict different ratios (phytoplankton reads∶total number of reads) and the points represent the samples.

After quality filtering and preprocessing 1,116 833 reads were obtained from the 259 sequenced samples included in the study, whereof nine percent or a total of 89,982 reads could be assigned to cyanobacteria or chloroplasts (from this onwards termed phytoplankton). The sequencing effort was highly variable among the samples ranging from 106 to 32,832 total reads per sample. Heterotrophic bacteria usually occur in higher numbers than phytoplankton, which is reflected in the ratio between phytoplankton reads and the total number of reads. This ratio was on average 0.098 (range from 0 to 0.58) and a distribution as depicted in Figure 1A. Low ratios together with low sequencing effort caused the number of phytoplankton reads to be too low to resolve the alpha diversity of the phytoplankton in most samples (see Figure 1B). To diminish the limitations of small sampling sizes for analyses on beta diversity and taxon dynamics, samples with less than 100 phytoplankton reads were removed, leaving 139 samples (54% of all samples) and a total of 82,825 phytoplankton sequences. The 139 selected samples represent lakes with a concentration range in total phosphorus from 2.9 to 149 µg L^−1^, total nitrogen from 0.4 to 1900 µg L^−1^, chlorophyll *a* from 0 to 40 µg L^−1^ and dissolved organic carbon from 2 to 32 µg C L^−1^ (see Table 1 for list of lakes used for analyses and Table S1 for associated metadata).

**Table 1:** Summary statistics of sequencing data including coordinates and classification of systems.

Lake	Lake type	#samples	#reads	#phyto reads	#OTUs	#phyto OTUs	Longitude	Latitude	reference
Lake Abraxas	antarctic	2	27652	5884	393	35	78.3	−68.5	Logares et al. 2012
Ace Lake	antarctic	1	31835	2121	2540	30	78.2	−68.5	Logares et al. 2012
Alinen Mustajarvi	dysotrophic	19	65380	3822	2133	166	25.1	61.2	Peura et al. 2012
Alstasjon	eutrophic	1	15612	739	860	49	12.0	63.0	Severin et al.
Atvandtjarnen	oligotrophic	1	30666	1693	1726	91	12.0	63.0	Logue et al. 2012
Bodsjon	oligotrophic	1	31330	1469	1748	119	15.4	62.8	Logue et al. 2012
Bredsjon	mesotrophic	1	3016	1226	225	94	13.9	61.8	Severin et al.
Bustadtjarnen	oligotrophic	1	18987	661	278	72	12.7	63.6	Logue et al. 2012
Crooked Lake	antarctic	1	31333	1971	1246	23	78.2	−68.6	Logares et al. 2012
Digernastjarnen	oligotrophic	1	5671	1951	246	104	12.7	63.6	Logue et al. 2012
Lake Druzhby	antarctic	1	2819	491	188	18	78.3	−68.6	Logares et al. 2012
Erken	mesotrophic	49	75173	11050	2269	196	18.6	59.8	Eiler et al. 2012
Fibysjon	mesotrophic	1	15610	109	788	39	17.4	59.9	Severin et al.
Funbosjon	eutrophic	1	30221	624	952	59	17.9	59.9	Severin et al.
Gravatjarnen	oligotrophic	1	30391	1273	939	95	12.3	63.6	Logue et al. 2012
Haggsjon	oligotrophic	1	30334	1774	1330	97	12.7	63.5	Logue et al. 2012
Hallastjarnen	oligotrophic	1	7641	1621	265	93	12.6	63.5	Logue et al. 2012
Lake Hand	antarctic	1	31348	1702	968	25	78.3	−68.6	Logares et al. 2012
Hassellasjon	dysotrophic	1	728	118	215	38	16.1	62.1	Comte et al.
Hensjon	oligotrophic	1	7846	1604	190	85	15.1	56.5	Logue et al. 2012
Highway Lake	antarctic	1	30564	853	1024	18	78.2	−68.5	Logares et al. 2012
Holmtjarnen	oligotrophic	1	25213	1589	1562	89	12.2	62.5	Logue et al. 2012
Lang-Bjorsjon	oligotrophic	1	31242	824	1096	83	12.3	63.6	Logue et al. 2012
Langsjon	mesotrophic	1	2857	311	386	40	17.6	60.1	Severin et al.
Lille Jonsvatn	oligotrophic	1	1617	162	213	33	10.6	63.4	Comte et al.
Lotsjon	mesotrophic	1	14967	226	867	23	18.0	59.9	Severin et al.
Marine Coastal site	antarctic	1	10136	1175	223	30	77.9	−68.6	Logares et al. 2012
Lake McNeil	antarctic	2	41432	4611	1171	33	78.4	−68.5	Logares et al. 2012
Medstugusjon	oligotrophic	1	8025	2373	313	114	12.4	63.6	Logue et al. 2012
Norrsjon	eutrophic	1	3605	451	316	36	18.0	59.9	Severin et al.
Organic Lake	antarctic	2	32895	2043	473	6	78.2	−68.5	Logares et al. 2012
Oster-Noren	oligotrophic	1	24734	2192	262	90	12.8	63.4	Logue et al. 2012
Ovre Langsjon	eutrophic	1	3446	374	296	61	18.0	59.9	Severin et al.
Pendant Lake	antarctic	2	18187	6662	591	43	78.2	−68.5	Logares et al. 2012
Ramsjon	mesotrophic	1	2922	626	221	33	17.5	59.8	Severin et al.
Rimov	mesotrophic	17	14894	3396	1958	203	14.5	48.8	This study
Rookery Lake	antarctic	1	27224	438	1029	12	78.1	−68.5	Logares et al. 2012
Ryssjon	eutrophic	1	3144	1147	339	93	17.2	59.8	Severin et al.
Lake Shield	antarctic	1	666	350	132	14	78.3	−68.5	Logares et al. 2012
Siggeforasjon	dysotrophic	1	1997	242	221	38	17.2	60.0	Severin et al.
Skalsvattnet	oligotrophic	1	18177	1505	198	101	12.2	63.6	Logue et al. 2012
Strandsjon	mesotrophic	1	3251	1174	443	69	17.2	59.9	Severin et al.
Tannsjon	oligotrophic	1	15094	1546	1933	89	12.7	63.4	Logue et al. 2012
Valloxen	eutrophic	1	2586	743	259	30	17.8	59.7	Severin et al.
Vaster-Noren	oligotrophic	1	6967	1746	149	99	12.8	63.5	Logue et al. 2012
Vereteno Lake	antarctic	1	8358	1344	326	20	78.4	−68.5	Logares et al. 2012
Lake Watts	antarctic	1	9292	467	194	18	78.2	−68.6	Logares et al. 2012
Lake Williams	antarctic	2	9694	1234	294	22	78.2	−68.5	Logares et al. 2012
Zurich	mesotrophic	4	1962	1118	401	35	8.8	47.2	This study

**Figure 2:**
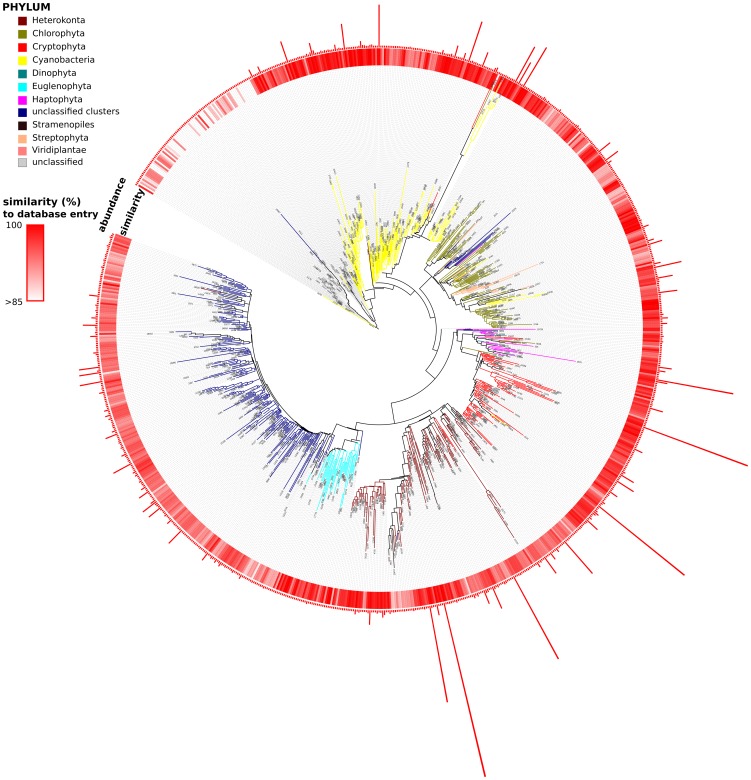
Phylogenetic tree (based on SILVA106 reference tree) showing representative sequences from all phytoplankton-related OTUs. Inner ring indicates the similarity of sequences to the nt/nr database (NCBI) as determined by BLAST search. Outer ring (bars) indicates the number of reads assigned to each node when using the resampled dataset (100 reads); note that nodes where all reads were removed by resampling are still given. Colored branches indicate group assignments from Bayesian classifier against a phytoplankton database.

For each of these 139 samples, the average number of reads annotated as cyanobacteria and chloroplasts was 596. This is in the same range as the average number of cells counted and classified by microscopy (at least 500) [34]. In total 946 phytoplankton OTUs were identified using the NGS based approach with an average 33.8 OTUs in each sample (range 4 to 117). Overall, Heterokonta was the most abundant phylum (28.3% of the reads), followed by Cyanobacteria (21.0%), Cryptophyta (18.3%), Chlorophyta (6.2%), Dinophyta (5.7%). Other phyla including Euglenophyta, Haptophyta and Streptophyta contributed less than 1% each. In addition, 16% of the reads were annotated to an unclassified sequence cluster, from now on termed, USC. The twelve taxa, with the highest proportion of reads in the dataset were (in order of their abundances) annotated as *Thalassiosira* sp. (Heterokonta), *Plagioselmis* sp. (Cryptophyta), *Cryptomonas* sp. (Cryptophyta), *Aulacoseira* sp. (Heterokonta), *Dinophysis*-related (most likely *Peridinium* and *Ceratium*; Dinophyta), *Cyanobium* sp. (Cyanobacteria), *Heterosigma-related* (most likely *Gonyostomum*; Raphidophyceae, Heterokonta), and *Microcystis* sp., *Synechococcus* sp. and *Prochlorococcus* sp. (all Cyanobacteria; for more detail see Figure 2).

To obtain the position of the USC reads in a phylogenetic framework, sequences were aligned and inserted into the SILVA106 phylogenetic tree. This analysis showed that the USC sequences form a deeply-branching sequence cluster and fall outside previously characterized entries (see Figure 2), but close to Euglenophyta. A Blastn search against the nr/nt databases further corroborate that USC belong to a so far uncharacterized group of photosynthetic eukaryotes at least by 16S rRNA gene standards and is related (up to 95% sequence similarity) to recently amplified single cell genomes of marine protists reported by Martinez-Garcia et al. [35].

### System comparison

**Figure 3:**
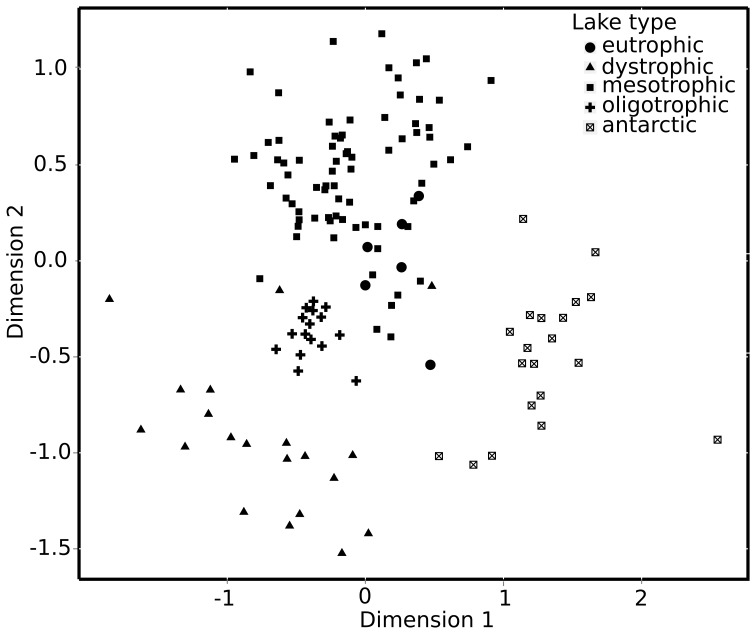
Ordination plot showing phytoplankton community composition among lakes of different trophic status (oligotrophic, mesotrophic, eutrophic and dystrophic). Stress value was 0.20. Permutational ANOVA confirmed visual inspection of significant differences in community composition between lakes of different status (p<0.001; R^2^ = 0.254).

Among the lakes, cyanobacterial reads dominated in samples from eutrophic systems (45.5%) and were also abundant in oligotrophic lakes (36.0%), while these lakes also featured a high proportion of USC reads (43.4%). Other OTUs affiliated with the USC dominated in humic lakes (32.9%) and were accompanied by almost equal relative amounts of reads (approx 12%) annotated to Chlorophyta, Cryptophyta, Cyanobacteria and Heterokonta. In samples from mesotrophic lakes most reads were annotated to Heterokonta (30.1%), Cyanobacteria (23.5%) and Cryptophyta (22.0%). Analysis of phytoplankton community composition by ordination of NGS data confirmed the clear differences described above in phylum composition among systems (see Figure 3). Here, oligotrophic lakes were in the center of the ordination and the other systems were clustered around these nutrient poor systems. Antarctic lakes were clearly different from all others, probably as a result of their saline character and possibly also their geographic location at high latitudes. Disparity between lakes of different trophic status was shown to be significant by permutational MANOVA (*p*<0.001; *R^2^* = 0.246; *pseudo-F* = 10.861). Posthoc pair-wise comparisons confirmed differences among lake types with mesotrophic and eutrophic lakes showing the least pronounced separation from each other (Table 2 and Figure 3).

**Table 2:** Results of permutational MANOVA comparing the phytoplankton communities among systems with different trophic status.

	antarctic			oligotrophic			mesotrophic			eutrophic		
	Fstats	R2	p	Fstats	R2	p	Fstats	R2	p	Fstats	R2	p
oligotrophic	22.14	0.39	>0.001									
mesotrophic	17.36	0.16	>0.001	13.62	0.13	>0.001						
eutrophic	8.48	0.26	>0.001	8.71	0.3	>0.001	2.56	0.03	>0.007			
dysotrophic	10.37	0.21	>0.001	9.71	0.22	>0.001	10.23	0.1	>0.001	3.89	0.13	>0.001

### Comparison of methods

Seasonal dynamics were analyzed in three lakes using both NGS and microscopy. Samples with both microscopic and NGS data available were 14 for AM, 34 for ER and 16 for RI. Using microscopy the total number of taxa were 58 in AM, 84 in ER and 107 in RI (see Table S2 for a detailed list); the average number of taxa in a sample was 25.5 with a range from 11 to 45. Analyzing the corresponding resampled samples from NGS revealed a total number of 102 OTUs in AM, 122 OTUs in ER and 140 OTUs in RI; on average 20 OTUs per sample were detected with a sampling effort resampled to 100 reads.

**Figure 4:**
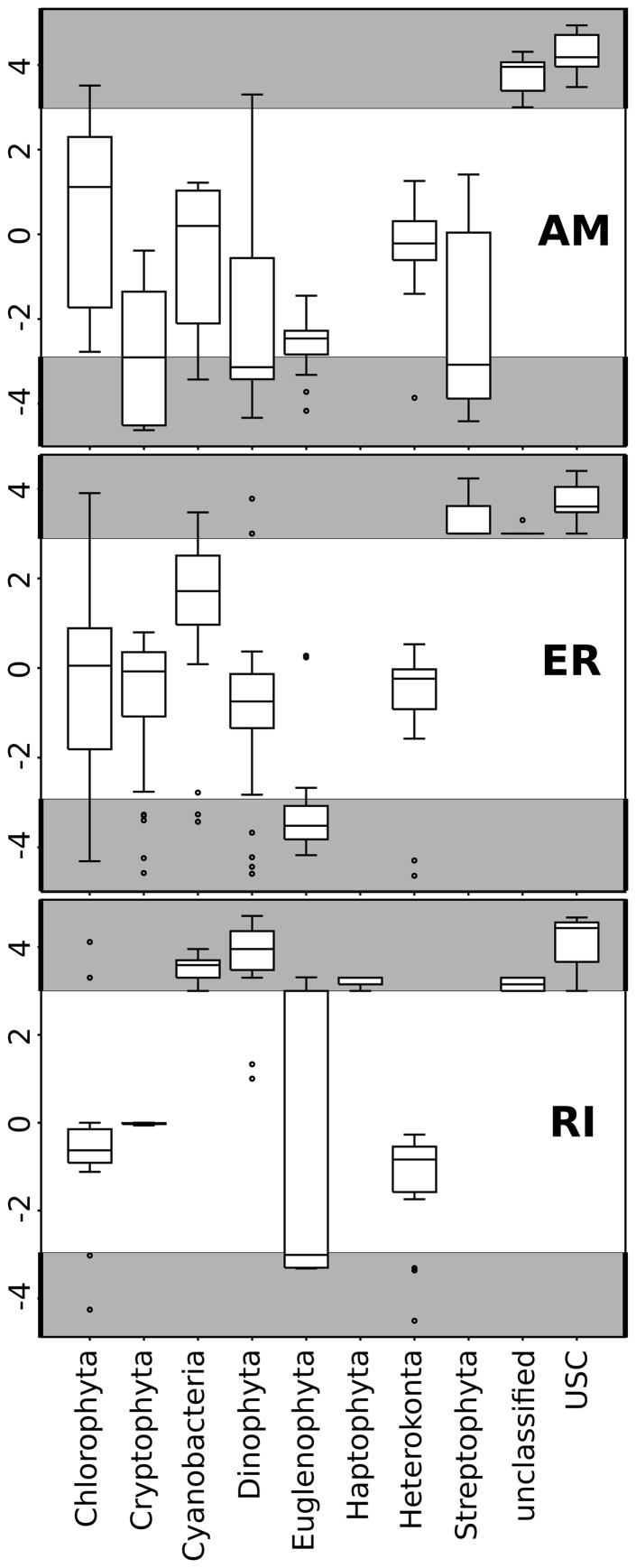
Boxplot showing ratios in taxonomic composition (at phylum level) as revealed by next generation sequencing (NGS) vs. microscopy. Plots show the ratio between relative reads numbers and biovolumes (as determined by microscopy) for each phylum. (AM) Alinen Mustajarvi, (ER) Lake Erken, and (RI) Rimov Reservoir. A ratio above zero indicates that a specific phylum is preferentially detected by NGS whereas a ratio below zero indicates an over representation in the biovolume data relative to NGS. The part of the plot indicated in grey represents the area where the ratio is the result of that a phylum was only detected by either method.

Statistical comparisons of seasonal phytoplankton dynamics in the three lakes (AM, ER, RI) by, on the one hand, cell abundance and biovolume data from microscopic counts and, on the other hand, NGS derived read numbers, revealed significant correspondence in the dynamics of community composition between the two methods, especially between microscopic abundance and NGS data. Here, both Procrustes superimposition and Mantel's test were significant (Table 3). Biovolume data showed a lower correspondence with NGS data and was not significant for RI. The correspondence of methods was less clear when comparing taxonomic groups in more detail (Figure 4). Heterokonta, Euglenophyta, Cryptophyta and Dinophyta were overrepresented in the microscopic biovolume data set compared to the NGS data, RI being an exception. A noteworthy 15% of the reads were annotated to USC, which was detected by NGS in all three lakes but was either missed or misclassified by microscopy. Cyanobacteria were proportionally overrepresented in the NGS dataset when compared to microscopic biovolume data (17.7% and 1.7%, respectively). Additionally, Dinophyta, a major phylum in the microscopic data, was only once detected by NGS in AM whereas it was regularly observed under the microscope. For ER, the taxonomic profiles corresponded well except for USC and Streptophyta, which were not detected by microscopy and Euglenophyta, which was not detected by NGS. In RI, only Dinophyta, Heterokonta, Cryptophyta and Chlorophyta were detected by both methods; whereas the other phyla were only detected by NGS.

**Table 3:** Results from Procrustes superimposition and Mantel's test to test for correspondence among methods.

	mantel's test		procrustes superimposition	
Testing 454 data against	R	p	R	p
AM biovolumes	0.259	<0.013	0.851	<0.012
AM abundances	0.26	<0.007	0.89	<0.005
ER biovolumes	0.268	<0.001	0.756	<0.001
ER abundances	0.532	<0.001	0.842	<0.001
RI biovolumes	0.083	0.289	0.617	0.371
RI abundances	0.654	<0.001	0.922	<0.001

**Figure 5:**
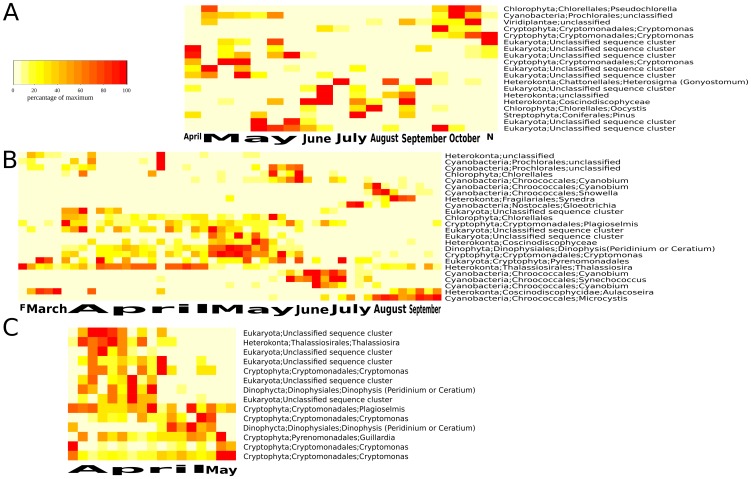
Heatmap showing temporal dynamics of phytoplankton taxa in dystrophic Lake Alinen Mustajärvi (A), mesotrophic Lake Erken (B), and mesotrophic reservoir Rimov (C) as revealed by next generation sequencing. Colors indicate the abundance of each taxon at each time point in relation to its maximum abundance in the respective time-series.

Looking at the dynamics in greater detail revealed further discrepancies but also correspondence between microscopy and NGS data. In AM, high abundance of Cryptophyta belonging to the genus *Cryptomonas* was observed from early spring to the late summer in the NGS data (Figure 5). An increase in the proportion of diatoms (Heterokonta) during late summer and their high abundance in late autumn was observed, whereas Chlorophyta and Cyanobacteria were negligible in this lake. The microscopic analysis showed a different pattern. Chlorophyta and Heterokonta (especially chrysophytes) were dominant during all seasons. Most other groups, including Cyanobacteria, were scarce. Similar to the NGS, microscopy revealed that Cryptomonas sp. was an abundant taxon and present in 93% of the lake samples. The other dominate taxa in the microscopy dataset were (in order of their abundance) *Oocystis* sp, *Scourfieldia cordiformis* (Chlorophyta), *Chrysococcus* sp., *Pseudopedinella* sp., *Monomastix* sp. (Heterokonta), *Koliella longiseta*, *Monoraphidium* sp., *Chlamydomonas* sp. (Chlorophyta), *Rhabdoderma* sp. (Cyanobacteria), *Uroglena* sp., *Mallomonas lychenensis*, (Heterokonta) and *Gymnodinium* sp. (Dinophyta). Note also that the NGS approach was able to pick up sequences from pollen of the tree *Pinus* (Figure 5a). Pollen were commonly found but not counted in phytoplankton analyses based on microscopy.

For ER, the NGS data showed that the succession started with a *Cryptomonas* bloom after ice-off immediately followed by a diatom bloom in spring (Figure 5b). Later during the season, a *Gloeotrichia* bloom was observed in July followed by a *Microcyctis* bloom in autumn. NGS data also indicated a high proportion of various putative single celled picocyanobacteria during the decline of the spring peak (June/July; Figure 5), which was overlooked in the microscopic analyses. Otherwise the NGS patterns were confirmed by the microscopy data as: Heterokonta were important in spring (mainly diatoms); bloom forming Cyanobacteria dominated in summer and autumn; Cryptophyta and chrysophytes (Heterokonta) were abundant groups throughout the year. The most abundant taxa based on microscopy were *Aphanocapsa* sp. (Cyanobacteria). Other abundant Cyanobacteria were *Aphanocapsa elachista* and *Coelosphaerium kuetzingianum*. Unidentified chrysophytes were also abundant as were *Chrysococcus* sp., *Aulacoseira granulata var. angustissima* and *A. islandica*, *Asterionella formosa* and *Dinobryon* sp. all from the group Heterokonta. *Cryptomonas* sp. and *Rhodomonas* sp. were abundant cryptophytes, and *Chrysochromulina parva* from the group Haptokonta were also among the most abundant taxa in this lake.

For RI, the peak of *Chlamydomonas* sp. under ice, as shown by NGS (Figure 5c), was also confirmed by microscopy. *Chlamydomonas* sp. was then replaced by Cryptophyta (*Rhodomonas minuta*, *Cryptomonas* sp.) and Chrysophyta (*Synura* sp., *Chrysococcus* sp.) and later in April by Haptophyta (*Chrysochromulina parva*) which form the spring maximum of biomass, as demonstrated by microscopy. The end of the sampling period was characterized by decreasing phytoplankton biomass dominated mostly by Cryptophyta. These complex patterns in Cryptophyta are reflected in the NGS data even though taxonomic assignments did not entirely correspond with that invoked by microscopic identification. Unidentified flagellates accounted for 0.3–17% of the total phytoplankton biomass, which could possibly be linked to the high presence of USC in RI as revealed by NGS.

## Discussion

Phytoplankton as primary producers, are directly using nutrients as a resource and are therefore early responders to environmental changes, making them especially suitable as eutrophication indicators. Our massive NGS dataset from 46 lakes revealed a clear separation of the phytoplankton communities from lakes of different trophy suggesting that this metric has potential as a tool for water quality status assessments. Thus, providing the means to efficiently monitor one of the main environmental problems in surface waters; eutrophication. Picophytoplankton are particularly useful as early indicators of increase in phosphorus concentration [36], [37] for marine as well as freshwater systems [38]. These small and often fragile organisms could be tracked and taxonomically highly resolved using the NGS based approach. It is also encouraging that seasonal patterns revealed by NGS data resembled well-described patterns from microscopy based observations in the three lakes where we had time series data (Table 3). Potential toxic cyanobacterial genera such as Gloeotrichia, Microcystis and Plankthotrix were resolved and tracked over time (Figure 5). To further track the frequency and intensity of toxic algal blooms, frequent sampling is imperative and this seems feasible with NGS based approaches.

### A critical view on the method

Rarefaction curves clearly show that our sampling efforts only scratched the surface of the phytoplankton diversity present in most studied systems. Increasing sampling efforts can provide a deeper insight into these communities, but this is limited by the actual proportion of phytoplankton 16S rRNA genes in the total pool of amplified 16S rRNAs in a sample. As visualized in figure 1A, the ratio of phytoplankton to total reads was above 1/20 (black slope) in 64% of the samples. Meaning that a sampling effort of at least 20,000 reads per sample in our study would have resulted in 64% of our samples having 1000 or more phytoplankton reads. By obtaining a sequencing depth of 100,000 reads per sample, the number of samples with 1000 or more phytoplankton reads would have increased to 94% (ratio 1/100 as represented by the red line). Aiming for 20,000 reads per sample will result in 98% of the samples having at least 100 phytoplankton reads as indicated by our dataset. Exactly how many reads per sample would be needed for robust estimates of trends in community composition and diversity among samples is not known with any certainty. We expect that this will be explored to a greater extent in coming publications, similar to other studies of bacterial diversity [39]. The importance of sampling depth when describing a community is, however, not a problem only in NGS based approaches, but is relevant also for microscopy based techniques. We expect that the potential for deep sampling is greater with NGS especially considering recent improvements in for example Illumina based sequencing technology [40].

The weaker correspondence of NGS data to microscopic biovolume estimates compared to abundances (Table 3) is likely explained by variations in the number of chloroplasts per cell (and corresponding number of 16S rRNA amplicons) since chloroplast numbers poorly reflect cell size [41], [42]. Further, a difference in taxonomic composition between NGS and morphological based data cannot be avoided (Figure 4). For NGS data, biases are introduced by the DNA extraction and PCR procedures [43], [44]. Underrepresentation of taxa in the microscopy samples can be because of 1) taxon-specific cell-losses during preservation or handling reported previously for protists [45]; 2) misleading or low resolution microscopic identification if cells are missing characters, for example akinetes for some Cyanobacteria, or flagella that may be lost or are hidden behind cells; 3) diatoms are almost impossible to discriminate based on morphological identifications without appropriate preparation; 4) as sedimentation chambers are commonly used, small cells that do not sink fast enough will be counted to a lesser extent or missed altogether. Thus, in summary, discrepancies between the two types of methods exist. Future research should seek to optimize and standardize all steps for an objective assessment of true diversity. For instance, the underrepresentation of certain taxa in some NGS samples (lake AM) can be partly explained by prefiltration with 50 µm sieves, excluding macrosized phytoplankton. Omitting this step is recommended in future studies.

Moreover, we are in the middle of revising the phylogeny of many phytoplankton groups. For example in diatoms [46], Cyanobacteria [47], [48] and green algae [49] paraphyletic and polyphyletic groups are found based on new genetic information. Comparisons of phenotypic (morphological) and genetic analyses are also hampered by contradictions between morphological and gene-based classification systems.

### Novel taxa and taxon resolution

Our analyses identified potential novel taxa and the lack of sequenced freshwater taxa in current databases. A BLASTn search revealed that more than 50% of the cyanobacteria and chloroplast reads in our dataset have no closely related neighbor (more than 97% similarity to a database entry) among 16S rRNA sequences from isolated phytoplankton strains (for more details see Figure 2). Many of the most abundant OTUs in our dataset were most closely related to database entries of marine phytoplankton (for example *Dinophysis*, *Heterosigma*, *Prochlorococcus*) which are well represented in 16S rRNA databases. This clearly shows that our current database does not cover most freshwater phytoplankton species. Even at a cutoff of 90% similarity, 1% of the reads were not similar to any sequence entry. In addition, recent efforts to sequence the microbes of the ocean has already revealed many phytoplankton taxa that have been previously missed by microscopy [9], [50], [51] and our study suggests that the same is most likely true also for lakes as indicated by the detection of USC. Barcoding of the cultured and characterized freshwater taxa have to be expanded before we can compare results from environmental surveys and can be sure about the existence of novel species or even phyla that have been missed so far.

Phylogenetic analysis also shows that taxonomic resolution provided by the 16S rRNA gene of chloroplasts can at best provides classification to the genus level. Another marker gene that has been used as a pre-marker for protists is the 18S rRNA gene [52] which provides superior resolution compared to the 16S rRNA gene of the plastids but at the cost of missing out on Cyanobacteria [8], [53]. We therefore suggest a hierachical approach by first targeting the variable region V3–V4 of the 16S rRNA gene as exemplified by this study. This reveals bacterial and most eukaryotic organisms with plastids using a single analysis and can then be coupled to a method with higher taxonomic resolution and deeper sampling of the eukaryotic (protist) diversity such as a 18S rRNA gene based second step analysis [54], [55]. Specific groups of protists can then be targeted with more specific markers providing high (maybe equal to species) resolution.

### Outlook

There is a need for improvement in environmental monitoring, both because of international regulations and because of public concern about blooms of toxic or nuisance algae and other environmental pressures. Our analyses suggest that NGS-based characterization of 16S rRNA genes hold great promise as tools for phytoplankton monitoring as it allows the simultaneous monitoring of bacteria and most eukaryotes with plastids in a high-throughput, reproducible and cost-efficient manner. Still, many challenges lay ahead before NGS based methods can be implemented in monitoring programs. Furthermore, NGS based approaches will of course only be semi-quantitative. Barcoding initiatives and thorough systematics using both genetic and morphological information will be required to improve sequence databases and existing taxonomic frameworks for tracking phytoplankton groups and monitor phytoplankton communities by NGS facilitated approaches. The use of alternative marker genes but also multiplexing need to be explored to improve taxonomic resolution. Most importantly, taxonomists and molecular biologists must come together and move the field forward to fully embrace and exploit NGS technologies for phytoplankton ecology and the quality management of inland waters.

## Supporting Information

Table S1 Metadata of 259 lakes available to this study including 139 samples used in the analyses of this study.
(XLS)

Table S2 List of taxa found from Lakes Alinen Mustajärvi (AM), Erken (ER) and Římov (RI) when analyzing phytoplankton samples by microscopy.
(DOC)
