# Supplementary material for: Unveiling Distribution Patterns of Freshwater Phytoplankton by a Next Generation Sequencing Based Approach
Source: PLoS One. 2013 Jan 22;8(1):e53516. doi: 10.1371/journal.pone.0053516 (PMC3551911; doi:10.1371/journal.pone.0053516)
Supplement: Table S2 — List of taxa found from Lakes Alinen Mustajärvi (AM), Erken (ER) and Římov (RI) when analyzing phytoplankton samples by microscopy. (DOC) [file pone.0053516.s002.doc]

Supplementary material xxx

List of taxa found when analysing phytoplankton samples in microscopy from Lakes Alinen Mustajärvi (AM), Erken (ER) and Římov (RI).

| **Taxa** | **AM** | **ER** | **RI** |
| --- | --- | --- | --- |
| Cyanobacteria |  |  |  |
| Chroococcales | x |  |  |
| Aphanocapsa sp. | x | x | x |
| Aphanocapsa elachista |  | x |  |
| Chroococcus limneticus |  |  | x |
| Chroococcus microscopicus | x |  |  |
| Chroococcus minutus |  | x |  |
| Chroococcus turgidus |  | x |  |
| Coelosphaerium kuetzingianum | x | x |  |
| Gomphosphaeria lacustris |  | x |  |
| Merismopedia minima |  | x |  |
| Merismopedia tenuissima |  | x |  |
| Microcystis sp. | x | x |  |
| Microcystis aeruginosa |  | x |  |
| Microcystis wesenbergii |  | x |  |
| Microcystis viridis |  | x |  |
| Snowella sp. | x | x |  |
| Woronichinia naegeliana |  |  | x |
| Nostocales |  |  |  |
| Anabaena sp. | x |  |  |
| Anabaena circinalis |  | x |  |
| Anabaena cylindrica |  | x |  |
| Anabaena lemmermannii |  | x |  |
| Aphanizomenon sp. |  | x |  |
| Gloeotrichia echinulata |  | x |  |
| Oscillatoriales |  |  |  |
| Leptolyngbya sp. |  |  | x |
| Oscillatoria sp. |  | x |  |
| Planktothrix agardhii |  |  | x |
| Pseudanabaena sp. |  | x |  |
| Pseudanabaena limnetica | x |  |  |
| Pseudanabaena mucicola |  | x |  |
| Romeria sp. | x | x |  |
| Synechococcales |  |  |  |
| Aphanothece sp. | x |  |  |
| Aphanothece clathrata |  | x |  |
| Cyanodictyon sp. | x |  |  |
| Rhabdoderma sp. | x |  |  |
| Synechococcus sp. |  | x |  |
| Heterokonta |  |  |  |
| Bacillariophyceae |  |  |  |
| Pennate diatoms |  |  | x |
| Achnanthes minutissima |  | x |  |
| Amphora perpusilla |  | x |  |
| Asterionella formosa |  | x | x |
| Cymatopleura sp. |  | x |  |
| Cymbella sp. |  | x | x |
| Diatoma elongata |  | x |  |
| Diatoma tenuis | x |  |  |
| Diatoma vulgaris |  |  | x |
| Entomoneis sp. |  | x |  |
| Fragilaria sp. | x |  |  |
| Fragilaria capucina |  | x |  |
| Fragilaria construens |  | x |  |
| Fragilaria crotonensis |  | x | x |
| Gomphonema sp. |  |  | x |
| Gyrosigma sp. |  | x |  |
| Meridion sp. |  | x |  |
| Navicula sp. |  | x |  |
| Nitzschia sp. |  | x |  |
| Nitzschia acicularis |  |  | x |
| Rhoicosphenia abbreviata |  | x |  |
| Surirella elliptica |  | x |  |
| Synedra acus |  |  | x |
| Synedra ulna |  | x | x |
| Tabellaria sp. | x | x |  |
| Tabellaria fenestrata var. actinastroides |  | x |  |
| Tabellaria flocculosa |  | x |  |
| Coscinodiscophyceae |  |  |  |
| Aulacoseira granulata |  | x |  |
| Aulacoseira granulata var. angustissima |  | x |  |
| Aulacoseira islandica |  | x |  |
| Aulacoseira italica |  |  | x |
| Cyclotella sp. |  |  | x |
| Melosira varians |  |  | x |
| Stephanodiscus sp. |  | x |  |
| Chrysophyceae | x | x |  |
| Bitrichia chodatii | x |  |  |
| Bitrichia phaseolus | x |  |  |
| Chromulina sp. |  | x |  |
| Chrysococcus sp. | x | x | x |
| Dinobryon sp. | x | x | x |
| Dinobryon borgei | x |  |  |
| Dinobryon divergens | x |  |  |
| Kephyrion sp. | x | x |  |
| Monochrysis parva | x |  |  |
| Ochromonas sp. |  | x |  |
| Stichogloea olivacea |  | x |  |
| Uroglena sp. | x |  |  |
| Uroglena americana |  |  | x |
| Dictyochophyceae |  |  |  |
| Pseudopedinella sp. | x |  |  |
| Raphidophyceae |  |  |  |
| Gonyostomum semen | x |  |  |
| Synurophyceae |  |  |  |
| Mallomonas sp. | x | x | x |
| Mallomonas caudata |  |  | x |
| Mallomonas lychenensis | x |  |  |
| Spiniferomonas sp. | x | x |  |
| Synura sp. |  | x | x |
| Xanthophyceae |  |  |  |
| Arthrodesmus trispinatus | x |  |  |
| Goniochloris fallax | x |  |  |
| Goniochloris mutica |  |  | x |
| Tetraedron regulare |  | x |  |
| Cryptophyta |  |  |  |
| Cryptomonas sp. | x | x | x |
| Cryptomonas curvata |  |  | x |
| Cryptomonas marssonii |  |  | x |
| Cryptomonas ovata |  | x |  |
| Cryptomonas reflexa |  |  | x |
| Planonephros parvula |  | x |  |
| Rhodomonas sp. |  | x |  |
| Rhodomonas lacustris |  |  | x |
| Rhodomonas lens |  | x |  |
| Rhodomonas minuta |  | x | x |
| Haptophyta |  |  |  |
| Prymnesiophyceae |  |  |  |
| Chrysochromulina parva |  | x | x |
| Dinophyta |  |  |  |
| Dinophyceae | x |  |  |
| Ceratium hirundinella |  | x |  |
| Gymnodinium sp. | x | x | x |
| Gymnodinium helveticum |  | x | x |
| Peridinium sp. | x | x | x |
| Peridinium aciculiferum |  | x |  |
| Peridinium inconspicuum | x |  |  |
| Peridinium umbonatum | x |  |  |
| Streptophyta |  |  |  |
| Closterium sp. |  |  | x |
| Closterium aciculare |  | x |  |
| Closterium acutum var. varibile |  | x |  |
| Cosmarium sp. | x | x | x |
| Elakatothrix sp. | x |  |  |
| Elakatothrix genevensis | x |  |  |
| Elakatothrix lacustris |  | x |  |
| Euastrum sp. | x |  |  |
| Mougeotia sp. | x |  |  |
| Staurastrum sp. | x |  | x |
| Staurastrum cingulum |  | x |  |
| Staurodesmus sp. | x |  |  |
| Staurodesmus extensus | x |  |  |
| Euglenophyta |  |  |  |
| Euglenophyceae | x |  |  |
| Astasia sp. | x |  |  |
| Euglena sp. |  | x | x |
| Trachelomonas sp. |  | x |  |
| Chlorophyta |  |  |  |
| Chlorophyceae | x |  |  |
| Chlorococcales |  |  |  |
| Ankyra sp. |  | x |  |
| Ankyra ancora |  | x | x |
| Coenochloris sp. |  | x |  |
| Coenocystis sp. |  | x |  |
| Micractinium pusillum |  |  | x |
| Neocystis policocca |  | x |  |
| Sphaeropleales |  |  |  |
| Ankistrodesmus sp. |  | x |  |
| Ankyra judayi sp. | x |  |  |
| Coelastrum sp. |  | x |  |
| Coelastrum astroideum |  |  | x |
| Coelastrum microporum |  | x | x |
| Crucigenia tetrapedia |  |  | x |
| Kirchneriella lunaris |  | x | x |
| Monoraphidium sp. | x |  |  |
| Monoraphidium arcuatum |  |  | x |
| Monoraphidium circinale |  | x |  |
| Monoraphidium contortum |  | x | x |
| Monoraphidium dybowskii | x |  |  |
| Monoraphidium minutissimum |  | x |  |
| Pediastrum boryanum |  | x | x |
| Pediastrum duplex |  | x |  |
| Planktosphaeria gelatinosa |  | x | x |
| Scenedesmus sp. |  | x | x |
| Scenedesmus acuminatus |  | x |  |
| Scenedesmus acutus |  | x |  |
| Scenedesmus ecornis |  | x |  |
| Schroederia robusta |  | x |  |
| Schroederia setigera |  | x |  |
| Tetraedron sp. |  |  | x |
| Tetraedron incus |  |  | x |
| Tetraedron minimum | x |  |  |
| Tetrastrum glabrum |  |  | x |
| Tetrasporales |  |  |  |
| Chlamydocapsa sp. | x |  |  |
| Pseudosphaerocystis lacustris |  | x |  |
| Sphaerocystis schroeteri | x | x |  |
| Volvocales | x |  |  |
| Carteria sp. |  | x | x |
| Chlamydomonas sp. | x | x | x |
| Chlorogonium sp. |  |  | x |
| Eudorina elegans |  | x | x |
| Gonium sociale |  |  | x |
| Pandorina sp. |  | x |  |
| Pleodorina indica |  |  | x |
| Volvox sp. |  | x |  |
| Nephroselmidophyceae |  |  |  |
| Nephroselmis angulata |  | x |  |
| Pedinophyceae |  |  |  |
| Scourfieldia cordiformis | x |  |  |
| Trebouxiophyceae |  |  |  |
| Botryococcus braunii |  | x |  |
| Chlorellales |  |  |  |
| Actinastrum hantzschii |  |  | x |
| Chlorella sp. |  | x |  |
| Closteriopsis sp. |  |  | x |
| Dictyosphaerium elegans |  | x |  |
| Dictyosphaerium primarium |  |  | x |
| Dictyosphaerium pulchellum |  | x |  |
| Koliella sp. |  | x |  |
| Koliella longiseta | x |  | x |
| Oocystales |  |  |  |
| Lagerheimia genevensis |  |  | x |
| Oocystis sp. | x | x |  |
| Oocystis marssonii |  |  | x |
| Oocystis solitaria |  | x |  |
| Ulvophyceae |  |  |  |
| Ulotrichales |  | x |  |
| Planctonema lauterbornii |  | x |  |
